# iBLAST: Incremental BLAST of new sequences via automated e-value correction

**DOI:** 10.1371/journal.pone.0249410

**Published:** 2021-04-22

**Authors:** Sajal Dash, Sarthok Rasique Rahman, Heather M. Hines, Wu-chun Feng

**Affiliations:** 1 National Center for Computational Sciences, Oak Ridge National Laboratory, Oak Ridge, TN, United States of America; 2 Department of Computer Science, Virginia Tech, Blacksburg, VA, United States of America; 3 Department of Biology, The Pennsylvania State University, University Park, PA, United States of America; 4 Department of Biological Sciences, The University of Alabama, Tuscaloosa, AL, United States of America; 5 Department of Entomology, The Pennsylvania State University, University Park, PA, United States of America; 6 Department of Electrical and Computer Engineering, Virginia Tech, Blacksburg, VA, United States of America; 7 Department of Biomedical Engineering and Mechanics, Virginia Tech, Blacksburg, VA, United States of America; 8 Health Sciences, Virginia Tech, Blacksburg, VA, United States of America; Universite de Lausanne Faculte de biologie et medecine, SWITZERLAND

## Abstract

Search results from local alignment search tools use statistical scores that are sensitive to the size of the database to report the quality of the result. For example, NCBI BLAST reports the best matches using similarity scores and expect values (i.e., e-values) calculated against the database size. Given the astronomical growth in genomics data throughout a genomic research investigation, sequence databases grow as new sequences are continuously being added to these databases. As a consequence, the results (e.g., best hits) and associated statistics (e.g., e-values) for a specific set of queries may change over the course of a genomic investigation. Thus, to update the results of a previously conducted BLAST search to find the best matches on an *updated* database, scientists must currently rerun the BLAST search against the *entire* updated database, which translates into irrecoverable and, in turn, wasted execution time, money, and computational resources. To address this issue, we devise a novel and efficient method to redeem past BLAST searches by introducing iBLAST. iBLAST leverages previous BLAST search results to conduct the same query search but only on the incremental (i.e., newly added) part of the database, recomputes the associated critical statistics such as e-values, and combines these results to produce updated search results. Our experimental results and fidelity analyses show that iBLAST delivers search results that are identical to NCBI BLAST at a substantially reduced computational cost, i.e., iBLAST performs (1 + *δ*)/*δ* times faster than NCBI BLAST, where *δ* represents the fraction of database growth. We then present three different use cases to demonstrate that iBLAST can enable efficient biological discovery at a much faster speed with a substantially reduced computational cost.

## Introduction

The utilization of a sequence similarity search tool is an indispensable step in most bioinformatics research involving nucleotide or protein sequences. *BLAST*, short for Basic Local Alignment Search Tool, is a widely used tool capable of conducting a sequence similarity search for a sequence of interest against a sequence database. BLAST relies on a heuristic approach for searching and provides results based on the identification of regions of similarity between target and query sequences through a seed-and-extend based local alignment [1]. The number of queries and the size of the reference database can significantly impact the execution time of BLAST.

Hence, the fast accumulation of sequences in NCBI-curated databases have a profound impact on the computational efforts required to perform sequence similarity searches. The sequencing data that is stored in the NCBI database has grown tremendously over the years, reportedly doubling the number of bases submitted to GenBank [2] every year over the last three decades (1982-present) (S1 Fig in the S1 File). This rapid accumulation of sequence data is one of the key factors responsible for transforming the field of genomics into one of the most demanding big-data science disciplines [3].

Given expanding amount of data, providing fast and biologically valuable sequence alignment tools via high-performance computing (HPC) and algorithmic innovations has been a highly active area of bioinformatics research, particularly in the context of rapidly expanding databases. For example, several sequence alignment programs have relied on contributing algorithmic improvements (e.g., HMMER [4], DIAMOND [5], CaBLAST [6]) while others have focused on improving parallelization to take advantage of emerging high-performance computing (HPC) platforms and programming paradigms (e.g., cuBLASTP [7], muBLASTP [8], mpiBLAST [9], SparkBLAST [10], and SparkLeBLAST [11]). Both DIAMOND [5] and CaBLAST [6] improve the execution time of sequence alignment by compressing the sequence database. Specifically, DIAMOND reduces the amino-acid alphabet while CaBLAST compresses the sequences by sequence redundancy. All of these sequence similarity search tools improve computational speed (i.e., reduce execution time) but sometimes at the cost of reduced sensitivity. DIAMOND only achieves 91%- 99% sensitivity [5] while CaBLAST achieves more than 99% sensitivity [6].

**Fig 1:**
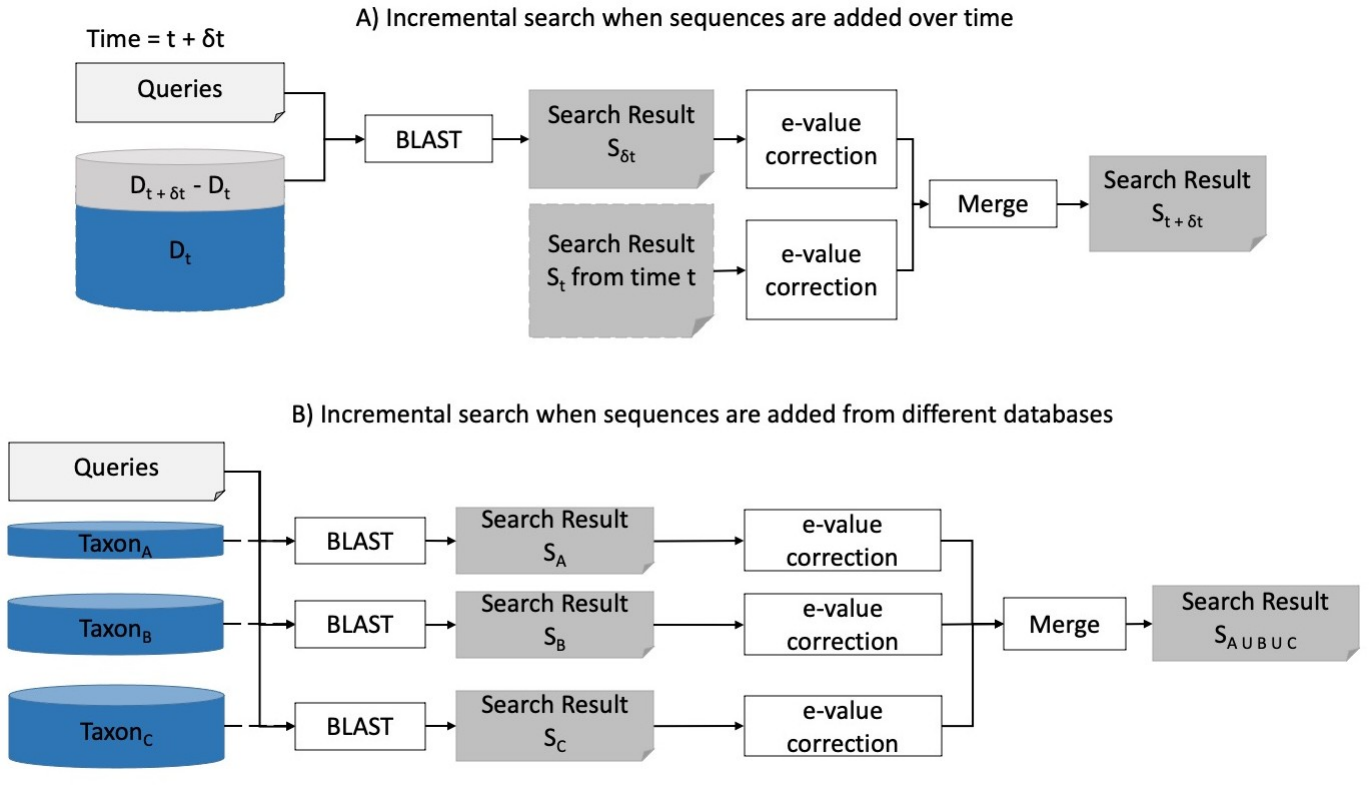
Addition of new sequences. (A) BLAST search when new sequences are added to the database. At time *t*, the database is *D*_*t*_. In next *δt* interval, new sequences *D*_*t*+*δt*_ − *D*_*t*_ are added, and the database becomes *D*_*t*+*δt*_. With the traditional approach, the prior search result at time *t* cannot be reused, and we have to perform an entire BLAST search against the entire *D*_*t*+*δt*_ database. (B) BLAST search when several taxon-specific databases are present and a result against the combined database is needed. For three taxa, A, B, and C, we can perform individual BLAST searches against the databases *D*_*A*_, *D*_*B*_, *D*_*C*_, respectively. If we want to obtain a search result against the combined database *D*_*A*∪*B*∪*C*_, we need to merge the search results in a way that their e-values reflect the combined database size.

Sequence similarity tools play a vital role in genome projects as annotations of assembled sequences require the utilization of BLAST-like tools for homology assignment. In reality, genome sequencing and annotation projects can be fairly long term, and thus, can require multiple sequence updates, e.g., regular annotation updates [12, 13]. However, such updates require executing sequence similarity search from scratch as BLAST uses similarity scores and e-values that depend on the ever-increasing size of the database. For this reason, it is currently required to discard the results of prior search efforts and rerun the entire search, which translates to irredeemable execution time, money, and computational resources. For bioinformatics projects requiring large-scale sequence similarity searches, such as those involving many transcriptomes from many taxa, the computational burden can be especially prohibitive. This problem could be addressed by performing iterative taxon-specific searches rather than conducting BLAST on the entire non-redundant (nr) database. However, adopting such an approach has the same problem of needing to standardize e-values while adding new databases to find the optimal identity of each query, as shown in Fig 1(B).

Currently, to the best of our knowledge, there exists no tool that can merge BLAST results of databases that have been added to either temporally (adding new searches over time) or spatially (combining results of two different searches).

We provide *a statistical approach to compose temporal and spatial BLAST search results through a novel method of e-value correction*. We derive how to do this mathematically and provide a software application called iBLAST to implement this automated e-value correction. By recycling previous BLAST search results, iBLAST provides substantial savings in execution time and computational resources since iBLAST performs (1 + *δ*)/*δ* times faster than NCBI BLAST, where *δ* represents the fraction of database growth. It enables taxon-specific BLAST searches, including incremental addition of searches of biologically-relevant taxa. The iBLAST tool consists of Python modules compatible with recent versions (since 2012) of NCBI BLAST command-line tools and can run on all major operating systems with minimal cognitive and installation overhead for NCBI BLAST users. This tool is thus especially useful for bioinformatics projects involving large-scale sequence search tasks. We demonstrate the efficiency and application of iBLAST using three case studies.

## Methods

To perform an iBLAST search *temporally*, we only need to consider the newly arrived sequences in the interval *δt* and perform a BLAST search against these sequences to get the result *S*_*δt*_, as shown in Fig 1(A). iBLAST then corrects the e-value scores for this incremental result *S*_*δt*_ and the past result *S*_*t*_ by using the size of the database *D*_*t*+*δt*_ = *D*_*t*_ + *D*_*δt*_. To perform an iBLAST search *spatially*, as shown in Fig 1(B), iBLAST examines the search results from different databases and corrects their e-values by using the size of the combined database *D*_*A*∪*B*∪*C*_ = *D*_*A*_ ∪ *D*_*B*_ ∪ *D*_*C*_. Then, iBLAST merges these search results with corrected e-values to obtain the final search result *S*_*A*∪*B*∪*C*_.

In the remaining part of this section, we present the details of our e-value correction methodology, the implementation details of iBLAST, and the fidelity and efficacy of iBLAST over NCBI BLAST via three case studies.

### BLAST concepts and statistics

#### Core concepts of a BLAST result: Hit, HSP, score, and e-value

When we perform a BLAST search against a sequence database with a query sequence, the BLAST program returns the sequences producing significant alignment from the target database, which we refer to as *hits*. Between the query and a hit sequence, there exist many pairwise locally-optimal gapped local alignments, which we refer to as *high scoring pairs* or *HSPs*. The definitions of hits and hsps are slightly different from those used by Althshul and colleagues [14], but follows the structural definition from the XML output format produced by NCBI BLAST. In the XML output format, the “Sequences Producing Significant Alignments” are presented as Iteration hits, and the “significant alignments” are listed as HSPs, though these are gapped alignments. One hit can consist of many HSPs. HSPs are scored using some statistical metrics when comparing aligned symbols. The score for a hit is the score of the highest-scoring HSP that belongs to that hit. The e-value for an HSP is computed using the score, the database size, and other statistical parameters. The reported e-value of a hit is the e-value of the highest-scoring HSP of this hit [15].

#### BLAST statistics for e-value computation

BLAST programs use two different types of statistics for e-value computation: Karlin-Altschul statistics and Spouge statistics. Both of these statistical formulae calculate e-value for the HSPs and hits. *blastn* and *tblastx* use Karlin-Altschul statistics while *blastp*, *blastx*, and *tblastn* use Spouge statistics.

**Karlin-Altschul statistics**. Karlin-Altschul statistics [1, 16, 17] measures the e-value using *E* = *e*^−λ(*S* − *μ*)^ = *Km*′*n*′*e*^−λ*S*^. This formula is adjusted for edge effect (see S1 File). Here *m*′ = *m* − *l*, *n*′ = *n* − *Nl*, *N* is the number of sequences in the database, *m* is the actual length of the query, and *n* is the actual length of the database.

The length adjustment *l* satisfies $l=\frac{\alpha }{\lambda }\ln\left((K(m-l)(n-Nl))\right)+\beta$. Here, *α*, *β*, *K*, and λ are statistical parameters.

**Spouge statistics**. Spouge statistics [18] is developed on the Karlin-Altschul formula. Instead of computing the length adjustment *l* and then using it to compute the effective length of the database and query, Spouge statistics applies a finite-size correction (FSC). Instead of estimating *l*, FSC estimates *area* = *E*[*m* − *L*_*I*_(*y*)]^+^[*n* − *L*_*J*_(*y*)]^+^ as a measure of (*m* − *l*)(*n* − *Nl*). The e-value *E* is then calculated as *E* = *area* × *Ke*^−λ*S*^ × *db*_*scale*_*factor* where $db\_scale\_factor=\frac{n^{\prime }}{m^{\prime }}$.

#### Existing e-value correction software and their features

mpiBLAST [9], a parallel implementation of NCBI BLAST on a cluster, partitions the database and performs BLAST searches against these partitions in parallel. For accurate e-value correction, mpiBLAST requires prior knowledge of the entire database [9]. NOBLAST [19] corrects e-values when split databases are in use and results need to be aggregated. However, it does not work with Spouge statistics. We provide a detailed explanation of these tools in Section “Existing e-value correction software and their features” in the S1 File. Both tools (mpiBLAST and NOBLAST) provide exact e-value statistics for Karlin-Altschul statistics when knowledge about the entire database is available *a priori*. However, they are not useful when the database keeps changing or when two different search results against two different instances of similar databases need to be aggregated. Table 1 provides a high-level comparison between mpiBLAST, NOBLAST, and our iBLAST.

**Table 1:** Comparison of three different BLAST tools that explicitly deal with e-value statistics correction. iBLAST supports e-value correction across time *and* space without requiring prior knowledge of the entire database while the other tools can perform e-value correction in limited scenarios.

Feature	mpiBLAST	NOBLAST	iBLAST
E-value correction for Karlin-Altschul statistics	✔	✔	✔
E-value correction for Spouge statistics	✘	✘	✔
Aggregate search results against pre-planned database segments	✔	✔	✔
Aggregate search results against arbitrary database instances	✘	✘	✔
Reuse existing search results	✘	✘	✔

#### Redundancy in data vs. redundancy in computation

Prior efforts to leverage redundancy in data (e.g., DIAMOND and CaBLAST) have successfully accelerated BLAST but at the cost of a small reduction in sensitivity. CaBLAST’s compressive algorithm achieves over 99% sensitivity for the improved speed [6]. Different versions of DIAMOND have sensitivity in the range 91.04% – 99% for various datasets [5]. In contrast, iBLAST aims to eliminate redundant computation while maintaining 100% sensitivity.

### e-value correction in an incremental setting

Correct e-value computation requires the actual database length (i.e., total number of bases/residues) in both Karlin-Altschul statistics and Spouge statistics. While database-partitioning parallel BLAST applications, like mpiBLAST and NOBLAST, require prior knowledge about the total database length, iBLAST leverages the partial knowledge from a previous BLAST search and combines it with the new sequence additions to the database to infer the total database length and compute the adjusted e-value in relation to the updated database. The mpiBLAST and NOBLAST tools pass the actual database length to each of their parallel jobs, thus forcing the statistics module to compute correct e-values from the beginning. For the iBLAST search, whenever new data arrives to the database, the pairwise sequence search is automatically refined in two steps. First, the search is only run on the databases constructed from *new* sequences that have been added to the database. Second, the results generated from searching the new sequences in the database are then merged with the saved results from the previous BLAST search.

#### e-value correction for Karlin-Altschul statistics

Let *n*_*c*_ represent the current database length and *n*_*d*_ represent the length of the newly arrived sequences for the database. Also, let *N*_*c*_ be the number of sequences in the current database and *N*_*d*_ be the number of sequences in the newly arrived part of the database. Then, we have

$$\text{Actual}\;\text{length}\;\text{of}\;\text{the}\;\text{updated}\;\text{database}:\;n_{t}=n_{c}+n_{d}.$$

$$\text{Total}\;\text{number}\;\text{of}\;\text{sequences}\;\text{in}\;\text{updated}\;\text{database}:\;N_{t}=N_{c}+N_{d}.$$

The actual query length *m* does not change with the change in the database. However, we do need to recompute the effective length *l* by solving the fixed-point equation for the new database length using Eq (1).

$$\begin{array}{c} l=\frac{\alpha }{\lambda }\ln(K\left(m-l\right)(\left(n_{c}+n_{d}\right)-\left(N_{c}+N_{d}\right)\times l))+\beta \end{array}$$ (1)

Now, with the updated length adjustment *l*, we can either recompute the e-values for all the matches or correct the e-values. To recompute all the e-values from scratch, we use Eq (2).

$$\begin{array}{c} E=e^{-\lambda (S-\mu )}=K\left(m-l\right)(\left(n_{c}+n_{d}\right)-\left(N_{c}+N_{d}\right)\times l)e^{-\lambda S} \end{array}$$ (2)

Alternatively, we can correct the e-values from the current values. First, we use *l* to recompute the value of the effective search space. We then use the newly computed effective search space to recalibrate the e-values for all the reported HSPs from the current and delta search results. Assuming that *D*_*part*_ is the partial effective search space and that *D*_*total*_ is the total effective search space, then the corrected e-value is given by

$$\begin{array}{c} E_{total}=E_{part}+Ke^{-\lambda S}\times \left(D_{total}-D_{part}\right) \end{array}$$ (3)

While both approaches require a constant number of arithmetic operations, the former approach, i.e., recomputing all the e-values from scratch, requires fewer arithmetic operations.

#### e-value correction for Spouge statistics

For Spouge statistics, the value of *area* described in Section “Spouge statistics” does not change since it is a function of the query length, sequence length, and Gumbel parameters. However, the database scale factor does change, and thus, we need to account for it. If the actual database lengths for the newly added part of the database and the total database are *n*_*part*_ and *n*_*total*_, respectively, then

$$\begin{array}{c} E_{part}=area\times e^{-\lambda S}\times \frac{n_{part}}{m}\;\text{and}\;E_{total}=area\times e^{-\lambda S}\times \frac{n_{total}}{m} \end{array}$$

So,

$$\begin{array}{c} E_{total}=E_{part}\times \frac{n_{total}}{n_{part}} \end{array}$$ (4)

Therefore, based on this derivation, we only have to re-scale the e-values instead of using Spouge’s e-value computational methods. Note: For re-scaling e-values that have been previously (and imprecisely) rounded to 0.0 by NCBI BLAST, re-scaling an e-value smaller than *e*^−180^ that was previously (and imprecisely) rounded to 0.0 by NCBI BLAST results in an incorrect 0.0 value. In this less than 0.1% occurrences of an extremely small but non-zero e-value, iBLAST ensures that this imprecise rounding does *not* occur.

### Merging two search results with correct e-value statistics

Once we correct e-values for both the current search result and the new search result, we merge the hits into a single sorted list. Because iBLAST reports some *better* scoring hits that NCBI BLAST misses (explained in detail in Section “iBLAST finds better scoring hits that are missed by NCBI BLAST”), reporting only *max*_*target*_*seqs* hits will result in missing some of the lower-scoring hits from NCBI BLAST. So, we store and report 2 × *max*_*target*_*seqs* hits. S3 Algorithm (in S1 File) documents the procedure to merge the hits from two results for the same query. All statistical parameters dependent on total database size are re-calibrated to recompute or re-scale the e-values. The hits are selected in the ascending order of their e-values (descending order of their scores). Additional details on recomputing and re-scaling e-values is provided in the S1 File (Section “e-value correction.”).

### iBLAST implementation

**Fig 2:**
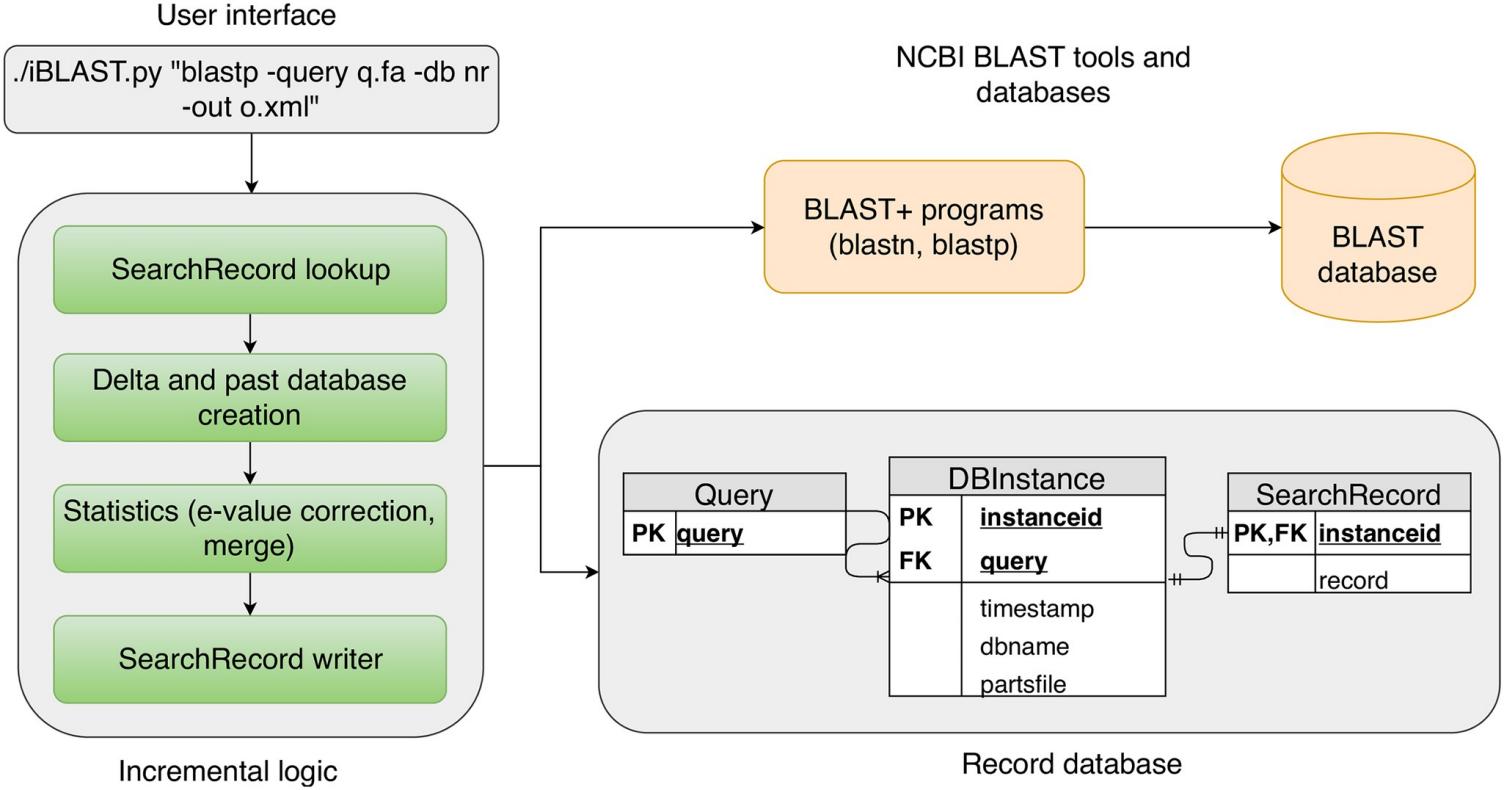
Software stack of iBLAST. The user can initiate a search using the user interface. The search parameters are then passed to the “Incremental logic” module. After performing an incremental search, this module’s back-end corrects the e-value statistics and merges the result. The “Incremental logic” module looks into an external lightweight database module called the (*Record database*) to decide whether and how to perform the incremental search. For the actual search and delta database creation, we use NCBI BLAST tools such as blastdbcmd, blastdbalias, blastp, and blastn.

We develop iBLAST for performing BLAST search as an extension to the NCBI BLAST code. It consists of Python wrapper scripts around the extended BLAST code and uses NCBI BLAST programs as black-box routines. Fig 2 shows the software stack of iBLAST, which consists of three major components: (1) user interface, (2) incremental logic, and (3) record database. These modules interact with BLAST databases through the BLAST+ programs.

#### Command-line user interface

In our current version, we provide a command-line user interface for iBLAST, which provides NCBI BLAST-like search options.

#### Incremental logic

This module decides whether to perform a new BLAST search based on current results. Whenever the user requests a new BLAST search, this module checks for any pre-existing search result.

If it does not find any pre-existing result, it performs a regular NCBI BLAST; but if there is a pre-existing result, the module first compares the database instance from the time of the past search with that of the present search. If there is any difference in the database size, this module builds a delta database that consists of the difference in these two instances. For computing the delta database, this module compares the lists of filenames of the two most recent instances and constructs a database alias using the difference (see Section “Computing delta database” in the S1 File for details). It then performs a new BLAST search only against the delta database and merges the previous result with the new incremental result after statistical correction for e-values. This module allows multiple updates to current searches with little extra time investment. The “Incremental logic” module contains four sub-modules: (1) SearchRecord lookup, (2) Delta and past database creation, (3) Statistics, and (4) SearchRecord writer.

1. ***SearchRecord lookup***. This sub-module looks for an existing search result with the help of the record database.
2. ***Delta and past database creation***. This sub-module constructs a delta database by comparing the current database against the database’s past instance and performs a BLAST search on the incremental database.
3. ***Statistics***. This sub-module reads the past and the new incremental search results; it then re-evaluates the e-values in both results and merges them according to their recomputed/re-scaled e-values.
4. ***SearchRecord writer***. This module writes the updated search result in one of the NCBI BLAST formats.

Whenever the user initiates a BLAST job, the above “Incremental Logic” module first checks if a current search result is available. A delta database consisting of the newly added sequences is constructed if there is a search result against an outdated BLAST database. A BLAST search is then performed against the delta database (i.e., incremental database). In the final stage, the e-values of the two search results are corrected and the incremental search results are merged. The frequency of this incremental search and re-computation depends on two quantities: the rate of database update by NCBI and the rate at which a research project performs a BLAST search.

**Record database for storing incremental search results**. Whenever the user performs a BLAST search, iBLAST saves meta-information (e.g., the size of the database and a list of the filenames) about the instance of the database and the search result in a lightweight SQLite database. We design iBLAST to save a minimalist index structure and size information that requires only a few bytes of storage. We keep the search parameters along with the search results as well. iBLAST does *not* save any redundant copy of any part of the actual sequence database. It stores only the most recent result for a specific query and a database, which keeps the storage overhead to a minimum.

### Case studies

To demonstrate the efficiency and benefits of using the iBLAST program over standard NCBI BLAST, we analyze different scenarios on actual nucleotide and protein sequence datasets as case studies. Our first case study tests the accuracy of iBLAST sequence searches compared to NCBI BLAST. The second case study assesses the value of adding new searches temporally to old ones for large, and the last case study assesses how this method can be used to add taxon-specific searches together to save on computational time.

#### Case study I: Method verification

We explore the scenario where hits from a collection of 100 query sequences are updated to account for the growth of NCBI sequence databases across the duration of the project. To demonstrate the application’s use for BLAST programs that use Karlin-Altschul statistics, we ran blastn against a nucleotide database (growing subsets of NCBI nt) for 100 nucleotide sequences from *Bombus impatiens* To demonstrate its utility on BLAST programs that use Spouge statistics, we ran blastp against a non-redundant protein database (a growing subset of NCBI nr) for 100 protein sequences from *Bombus impatiens* assembly. Source of these data is available at the S1 File (Section “Data source for case study I”).

**Fig 3:**
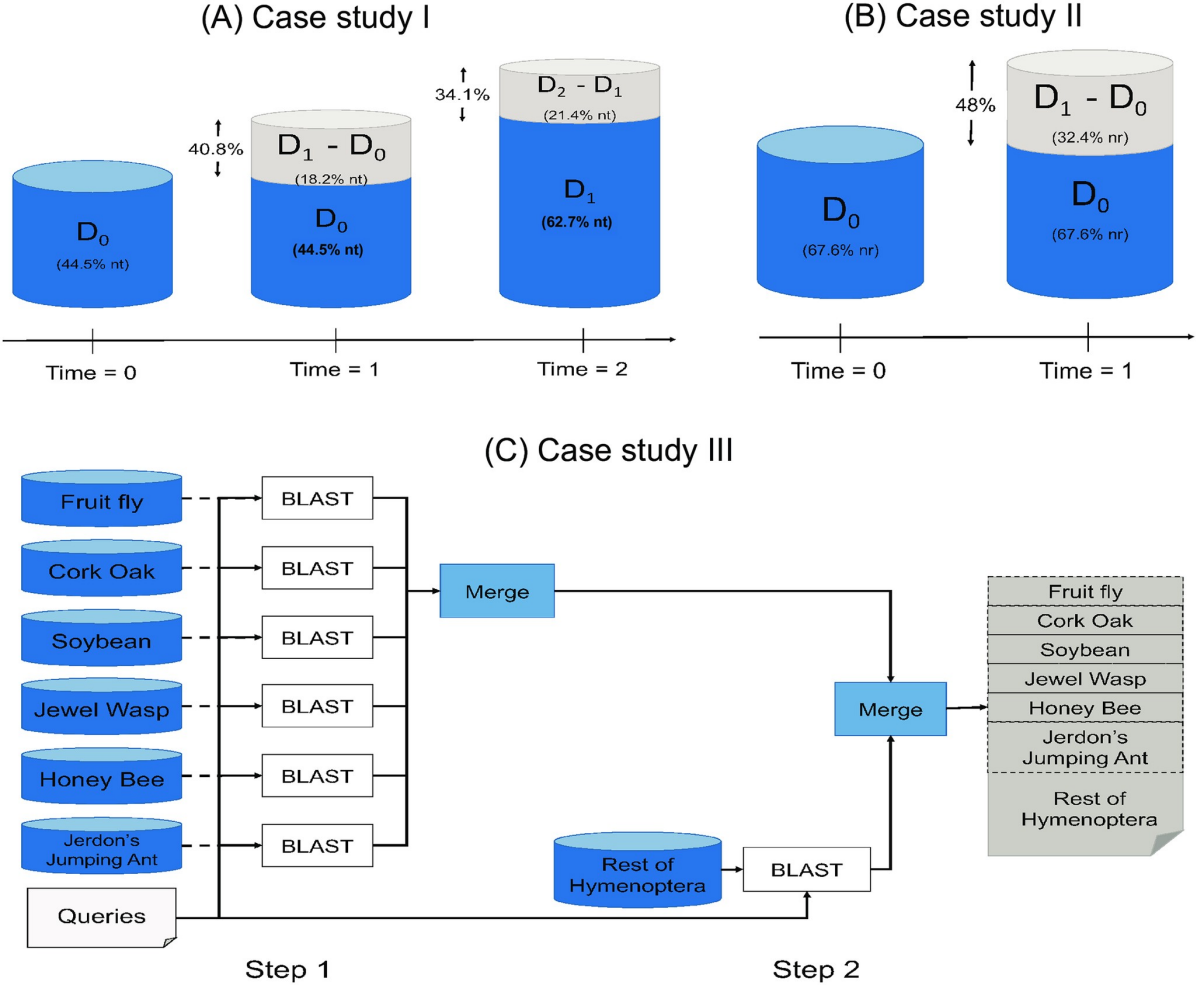
Experimental design of three case studies. (A) Case study I: Incremental addition of sequences in the nt database over three time periods. (B) Case study II: Incremental addition of sequences in the nr database over two time periods. (C) Case study III: Incremental search of taxon-specific databases.

We demonstrate iBLAST’s fidelity and performance over three time periods for case study I, as shown in Fig 3(A). The instances for nucleotide database changes through time as follows:

- **Time 0**: The nucleotide database comprises 44.5% of the fully available nt database. Both NCBI BLAST and iBLAST search on the same database.
- **Time 1**: The nucleotide database comprises 62.7% of the nt database. While NCBI BLAST searches 62.7% of nt, iBLAST searches only 18.2% of nt. The database grew by 40.8% (= (62.7 − 44.5)/44.5) from time 0.
- **Time 2**: The nucleotide database comprises 84.1% of the nt database. While NCBI BLAST searches 84.1% of nt, iBLAST searches only 21.4% of nt. The database grew by 34.1% (= (84.1 − 62.7)/62.7) from time 1.

For this case study, we also similarly applied NCBI BLAST and iBLAST to an evolving nr database. That is, the instances of the protein database change over time (in a similar way to the nt database, as captured by Fig 3(A)). Specifically, the nr database comprises 35.4%, 47.5%, and 60.0% of nr at times 0, 1, and 2, respectively. The protein database grew by 34.1% (i.e., (47.5 − 35.4)/35.4) and 26.3% (i.e., (67.5 − 35.4)/48) by times 1 and 2, respectively, from the earlier time periods. Table 2 provides detailed information about the evolving protein database instances as well as the e-value and hit performance of NCBI BLAST and iBLAST, respectively.

**Table 2:** Case study I: Fidelity of iBLAST in three consecutive time periods. *blastn* search was performed on nucleotide sequence databases (nt). At any time instance, the *Past* database size is the size of the database from the previous time instance. The *Present* database size is the database size at the present time instance. *Delta* is the incremental database growth from the previous time instance to the current time instance. NCBI BLAST must be performed on the entire *Present* database size, while iBLAST only needs to be performed on *Delta*.

				NCBI BLAST	iBLAST		
Time	Search	Data-base	Database Size		Delta = Present-Past	e-value Match	Hit Match
			Past	Present			
*t*_0_	blastn	nt	0	80,740,533,243	80,740,533,243	100%	100%
*t*_1_	blastn	nt	80,740,533,243	113,749,495,340	33,008,962,097	100%	100%
*t*_2_	blastn	nt	113,749,495,340	152,471,828,601	38,722,333,261	100%	100%

Additional details on the creation of the incremental database can be found in Section “Creating experimental databases” in the S1 File.

#### Case study II: Updating a query re-annotation of a novel transcriptomics dataset

Our second case study mimics a typical scenario in a transcriptome re-annotation project where a transcriptome is BLAST-ed after a certain period of time as a part of a re-annotation pipeline. This case study uses a novel dataset not yet available on the NCBI BLAST database—a *de novo* assembled transcriptome of the venom gland of an Oak gall wasp (see below)—and thus, the identity of the assembled sequence was unknown, and the sequence was not available to BLAST to itself.

As shown in Fig 3(B), we conduct a BLAST search for the same query set for database instances at two time instances (S3 Table in S1 File):

- **Time 0**: The database comprises 67.6% of the non-redundant database nr (nr accessed on August 2018). Both tools perform the search on this same 67.6% of the database.
- **Time 1**: The database comprises 100% of the non-redundant database nr. While NCBI BLAST performs a search on 100% of nr, iBLAST only needs to search 32.4% of nr as it can reuse the search results from time 0. iBLAST merges the result from time 0 with the incremental search result after e-value correction.

We constructed these two database instances by combining database parts using the blastdb_aliastool utility packaged with BLAST+.

Given the number of queries from the *de novo* assembled transcriptome, it would take a few *months* to complete the search on a single processor core. We ran this experiment with 640 cores distributed across 20 compute nodes (where each node contained dual 16-core Intel Xeon processors, i.e., E5-2683 v4), partitioning the 17, 927 queries into 20 query files and assigning each file per node. Given that each node would run a subset of queries against the same database, there is no need to recompute the statistics for these results before we merge them.

**Distributing workload across nodes**. The workload across all the nodes should be relatively balanced so that computation for each of the 20 query files finishes roughly simultaneously. To ensure such load balancing, we partitioned the queries using the following strategy. We randomize the order of the queries and partition them so that each partition has roughly the same number of residues. We compare this strategy against the straightforward approach of partitioning the queries in a linear order by putting roughly the same number of queries in each partition.

#### Case study III: Taxon-based incremental approach

Our third case study presents a special case of using a taxon-based incremental approach to obtain a fast, cost-effective, and biologically relevant results for sequence similarity. To achieve this goal, we examine the genes contained within an assembled transcriptome of the venom gland of a gall wasp of oak trees, the hedgehog gall wasp (*Acraspis erinacei*), a taxon lacking a closely related species with a genome in the nr database. Gall wasps are a group of parasitic wasps that inject their eggs into plant tissues and induce changes in plant development. These changes result in constructing a niche for the gall wasp by inducing predictable modifications of plant tissues that both protect the wasp from the environment and feed the developing wasp. Genes important for inducing changes in the plant’s development are thought to be produced in the female venom gland during oviposition [20]. We performed separate BLAST searches of the hedgehog gall wasp venom gland against transcriptomes of the closest relatives to gall wasps with curated genomes including three fairly equidistant taxa [21]—the parasitic wasp *Nasonia vitripennis*, the honey bee *Apis mellifera*, and the ant *Harpegnathos saltator*,—as well as the more distant model insect, *Drosophila melanogaster*, upon which many insect gene annotations are based. We also performed BLAST searches against the transcripts of an oak tree, *Quercus suber*, to determine if some genes belonged to the host as well as a model plant, the soybean, *Glycine max*.

Using iBLAST, we performed a blastp search individually against each of the databases and merged the results using the statistics module from iBLAST. After this initial search, we then added to this analysis all remaining Hymenopteran species using iBLAST to assess the impact of adding more taxa on the top BLAST hits and further demonstrate the potential of iBLAST to add taxa progressively, as shown in Fig 3(C). We performed a blastp search against those seven subsets’ merged database to determine whether the same hits would have been found from our concatenated incremental analysis as from a combined single-instance run. These results were further compared with blastp results obtained by searching the complete nr database, allowing us to determine how well we captured the full dataset with this taxon sub-sampling approach. Data collection methods for the gall wasp transcriptomes are provided in the S1 File (Section “Data collection for case studies II and III”).

## Results

We created a tool called iBLAST for e-value correction and incremental BLAST search in the temporal and spatial domain. The incremental search can be implemented using the software available at https://github.com/vtsynergy/iBLAST following instructions available in the S1 File (Section “iBLAST software allows the user to perform incremental BLAST search with minimal overhead”). The findings of the three case studies demonstrate the efficacy and utility of iBLAST.

### Case study I: Method verification and performance

#### Verification

In case study I, we validate whether we can achieve the same results from a single NCBI BLAST search as from the iBLAST. As shown in Table 2 and S4 Table in S1 File, iBLAST delivers the same results as NCBI BLAST with a 100% e-value match and 100% hit match.

- **blastn**: Sequence alignment using blastn was performed on nt databases (nucleotide sequences). In all three time periods, iBLAST finds all the same hits and in the same order as NCBI BLAST does for blastn, including 3, 964 hits at time *t*_0_; 4, 150 hits at time *t*_1_, and 4, 924 hits at time *t*_2_, thus validating iBLAST with respect to Karlin-Altschul statistics (Table 2).
- **blastp**: Sequence alignment using blastp was performed on nr (non-redundant protein sequences) databases. For each of these three time periods, iBLAST reports the same hits in the same order as NCBI BLAST for blastp. The numbers of reported hits in these three time periods for blastp are 45, 154 hits; 46, 356 hits; and 46, 869 hits, respectively, thus validating iBLAST with respect to Spouge statistics (S4 Table in S1 File).

#### Performance

**Fig 4:**
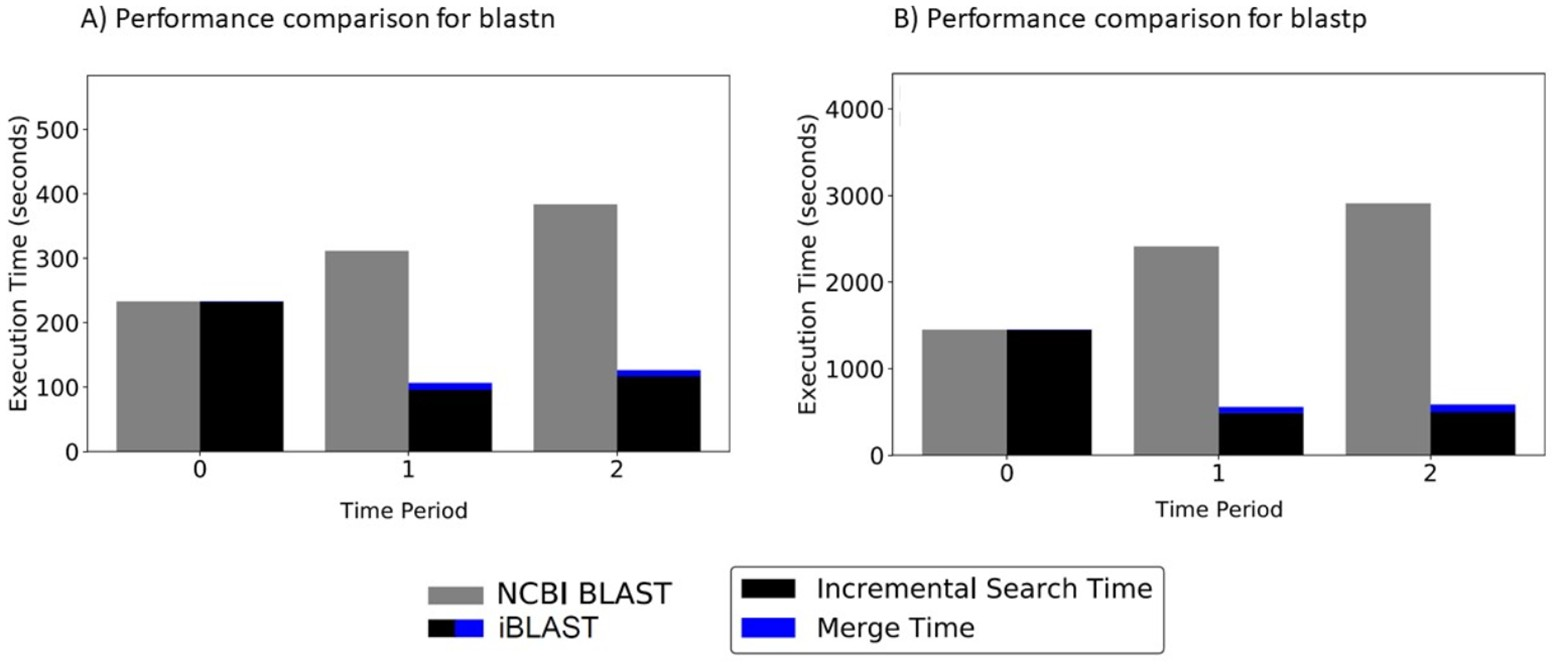
Performance comparison between NCBI BLAST and iBLAST for case study I. (A) Performance comparison between regular blastn and incremental blastn at 3 periods when nt database is growing over time, using 100 nucleotide queries. For 40.8% and 34.0% increase in the database size, iBLAST performs 2.93 and 3.03 times faster respectively. (B) Performance comparison between regular blastp and incremental blastp at 3 periods when nr database is growing over time, using 100 protein queries. For 34.1% and 26.3% increase in the database size, iBLAST performs 4.33 and 4.98 times faster respectively.

For a *δ* increase in database size, iBLAST performs (1 + *δ*)/(*δ*) times faster than NCBI BLAST. Fig 4 shows the time saved for both blastp and blastn, respectively, using iBLAST, resulting in a speedup ranging between approximately three- and five-fold.

### Case study II: Large-scale alignment tasks on novel datasets

We performed searches using iBLAST and NCBI BLAST, where a newly obtained gall wasp (Hymenoptera: Cynipidae) transcriptome dataset was utilized as queries in two time periods across which there was a 48% increase in the nr database (S5 Table in S1 File). In both time periods, iBLAST reports the same hits in the same order as an NCBI BLAST run.

For this increase in the database size, iBLAST is 3.1 times faster than NCBI BLAST. Relative to the total execution time of 134 minutes, the time needed for e-value correction and merging the results is minimal, i.e., less than a minute using only 20 cores. Overall, NCBI BLAST completes the alignment search in 24, 862 seconds (6 hours, 54 minutes) on average, while iBLAST completes the search in only 8, 009 seconds (2 hours, 14 minutes). The merge time for each of these tasks is 40 seconds on average. This computational efficiency matches our projected speedup (1+ 0.48)/0.48 = 3.08.

We observed the effect of query partitioning on load balancing (see Section “Distributing workload across nodes”). Our approach to partition the queries based on the number of residues shows superior load-balancing over the traditional strategy to partitioning the queries based on the number of queries. We elaborate on this point further in the S1 File (Section “Load-balancing via query partitioning”).

### Case study III: Taxon-specific searches to expedite informatics

To examine the fidelity of iBLAST while merging multiple (taxon-specific) databases, we first compared the iBLAST merged results from multiple individual BLAST (blastp) searches on seven biologically relevant taxa separately to results obtained when a BLAST search was performed against a database combining all the sequences belonging to these taxa simultaneously. The result exhibits 100% fidelity. Then, as presented in Table 3, we compare the merged BLAST results of individual taxon-level database search with the BLAST results obtained in case study II (time period 1), where the same queries were searched against the entire nr database to better understand the relative time savings vs. accuracy of taxon-guided approaches. The taxon-specific approach is much more time-efficient and computationally inexpensive as it searched much smaller-sized databases. 8.12% of the top hits obtained from a search of nr were found by searching only the initial set of 6 taxa, which comprise only 0.35% of nr. Although this number is low, the identity of high-scoring hits is likely to be similar even if the best taxonomic hit to a query sequence was not retrieved, as gall wasps do not have any close relatives currently hosted in NCBI databases but rather many equidistant relatives. Given this, we then added in sequences of the rest of Hymenopteran species to see if this improves the number of shared top hits. With this analysis, we conducted BLAST search on only 1.17% of the total nr yet obtained 87.75% similarity in top hits to a full nr BLAST. This result demonstrates the potential of performing more taxon-guided approaches to save on the costs of large-scale BLAST searching jobs. Performing the analysis in this way has also enabled improved curation of hits by taxon, which facilitates better biological interpretation of these results.

**Table 3:** Potential for taxon-guided searches enabled by iBLAST. Comparison of merged BLAST results from multiple individual BLAST searches with a separate BLAST search conducted against a completed nr database shows that biologically relevant taxa can be added incrementally to obtain similar results to nr by searching against a much smaller database size.

Species	NCBI taxon id	%nr sequences covered	Number of nr top hits covered	%nr top hits covered
*Nasonia vitripennis* (jewel wasp)	7425	0.02%	853	4.84%
*Apis mellifera* (honey bee)	7460	0.02%	207	1.17%
*Harpegnathos saltator* (Jerdon’s jumping ant)	10380	0.03%	347	1.96%
*Drosophila melanogaster* (fruit fly)	7227	0.08%	6	0.034%
*Quercus suber* (cork oak)	58331	0.09%	0	0.00%
*Glycine max* (soybean)	3847	0.11%	22	0.12%
Rest of Hymenoptera	7399	0.83%	14281	80.98%
**Total**	**multiple**	**1.17%**	**15476**	**87.75%**

### iBLAST finds better scoring hits that are missed by NCBI BLAST

While iBLAST finds all the hits reported by NCBI BLAST in the same order of appearance, iBLAST reports several *better scoring hits* that NCBI BLAST misses in all the case studies. Since case study II covers the most number of hits, we quantified these missed hits for this case study. NCBI BLAST misses 1.57% (13171 out of 837942 top hits) of the better scoring hits. Command-line NCBI BLAST uses a search parameter *max_target_seqs* in an unintended way where instead of reporting all the *best* *max_target_seqs* hits, it has a bias toward *first* *max_target_seqs* hits. A comprehensive discussion about this issue was carried out by Sujai Kumar (https://gist.github.com/sujaikumar/504b3b7024eaf3a04ef5/) and two other teams of researchers [22, 23]. In this process, it misses some of the better scoring hits that are discovered in a later phase of the search. (Details can be found in Section “Explanation for NCBI BLAST missing many top hits” of the S1 File) This is an extra advantage of iBLAST over NCBI BLAST. Since the former works on smaller databases and then combines the results instead of searching a single large database, it has more candidate hits to choose from for reporting final hits.

## Discussion

In this paper, we have introduced iBLAST, an incremental local-alignment tool that enables combining multiple search results with e-value correction. iBLAST delivers results that comprehend those of NCBI BLAST. Our statistical correction facilitates novel ways of performing sequence alignment tasks and incorporating domain knowledge. For a *δ* fraction increase in the database size, iBLAST can perform (1 + *δ*)/*δ* times faster than NCBI BLAST (i.e., 10% growth in database size will yield an 11-fold speedup for iBLAST over NCBI BLAST). We should note that for a small increase in the database size (which is the most likely scenario between two searches), iBLAST delivers a large speedup factor. Furthermore, iBLAST discovers better hits than NCBI BLAST. While iBLAST finds 100% of the hits that NCBI BLAST reports in the same order, iBLAST also reports many additional high-scoring hits that NCBI BLAST misses due to an early approximation used by the heuristic search algorithm in NCBI BLAST.

With the expansion of genetic (sequencing) data available in NCBI, the computational time for large-scale analyses becomes increasingly burdensome, resulting in analyses that take months to complete with a substantial cost, both financially and with respect to “time to solution.” This problem is aggravated by cheaper sequencing technology leading to ever-larger genome assembly/transcriptomics projects with substantially more samples to analyze. Our iBLAST tool can help relieve this cost burden. Utilization of iBLAST can enable frequent iterative updates for re-annotation of genome and transcriptome assemblies at a much lower cost (with respect to computational time and financial cost), which is useful given the rapid changes in the nr databases across the duration of a project or its aftermaths. We can add specific datasets of interest to previous searches, such as scenarios involving the availability of new genome releases or conducting large phylogenetic studies. As demonstrated in the final case study, we can use the program in transcriptomic or metagenomics projects by merging the results of knowledge-guided BLAST searches only on biologically relevant groups. The approach used in that case study enables iterative exploration by taxon and facilitates BLAST results’ curation.

Our iBLAST software can work as a wrapper around other fast BLAST implementations and provide multiplicative speedup on the wrapped applications’ speedup. iBLAST’s improved runtime performance is due to the incremental nature of its algorithm. So, it will be $\frac{1+\delta }{\delta }$ times faster for a *δ*-fractional growth compared to other non-incremental optimized implementations of BLAST such as cuBLASTP if the former is used as a wrapper around the latter. These other tools have varying degrees of sensitivity compared to NCBI BLAST. mpiBLAST [9] produces similar search results, CaBLAST’s compressive algorithm [6] achieves over 99% sensitivity for the improved speed, and various versions of DIAMOND have sensitivity in the range 91.04% − 99% for various datasets [5]. iBLAST achieves 100% sensitivity and can improve other tools’ sensitivity.

A similar approach can benefit other sequence similarity tools and their various implementations if the statistics for correcting the respective statistical significance values (analog to e-value) of the results are available. We aim to develop a standard pipeline for other popular sequence-similarity search tools to combine results through a framework for automated statistical correction in future work. Through its statistical correction formulas and software stack, iBLAST presents the potential to make other sequence similarity-search tools faster by utilizing past search results and incorporating domain knowledge in a period when sequence database is growing exponentially.

## Supporting information

S1 File (PDF)
