# Supplementary material for: iBLAST: Incremental BLAST of new sequences via automated e-value correction
Source: PLoS One. 2021 Apr 22;16(4):e0249410. doi: 10.1371/journal.pone.0249410 (PMC8062096; doi:10.1371/journal.pone.0249410)
Supplement: S1 File — (PDF) [file pone.0249410.s001.pdf]

iBLAST: incremental BLAST of new sequences via automated e-value correction

Sajal Dash[1,2*], Sarthok Rasique Rahman[3,4], Heather M. Hines[3,5], Wu-chun Feng[2,6,7,8*]

1 National Center for Computational Sciences, Oak Ridge National Laboratory, Oak Ridge, TN, USA

2 Department of Computer Science, Virginia Tech, Blacksburg, VA, USA

3 Department of Biology, The Pennsylvania State University, University Park, PA, USA

4 Department of Biological Sciences, The University of Alabama, Tuscaloosa, AL, USA

5 Department of Entomology, The Pennsylvania State University, University Park, PA, USA

6 Department of Electrical and Computer Engineering, Virginia Tech, Blacksburg, VA, USA

7 Department of Biomedical Engineering and Mechanics, Virginia Tech, Blacksburg, VA, USA

8 Health Sciences, Virginia Tech, Blacksburg, VA, USA

* dashs@ornl.gov, feng@cs.vt.edu

Supplementary material

Growth of sequence data

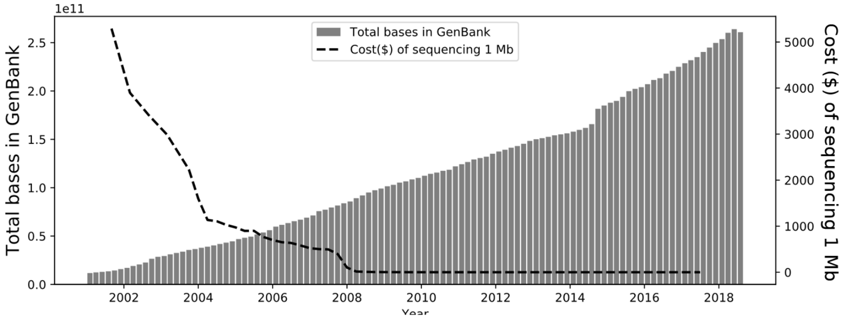

Fig S1. Growth of sequence database size compared to the sequencing cost. Increasing GenBank database size available at `https://www.ncbi.nlm.nih.gov/genbank/statistics/`, accessed on September 15, 2018) follows a decreasing trend in sequencing cost (available at `https://www.genome.gov/about-genomics/fact-sheets/DNA-Sequencing-Costs-Data`, accessed on September 15, 2018) .

BLAST Statistics for E-value computation

BLAST programs use two different kinds of statistics for e-value computation: Karlin-Altschul statistics and Spouge statistics.

Karlin-Altschul Statistics

Smith-Waterman local alignment scores between two random sequences follow the Gumbel (Type I) extreme value distribution (EVD). Under the extreme value theorem, the generalized extreme value distribution is the limit distribution of properly normalized maxima of a sequence of independent and identically distributed (i.i.d.) random variables. Therefore, the generalized extreme value distribution, including the Gumbel EVD, is often used to approximate the distributions of the maxima of long sequences of random variables, in this case, the distributions of the HSPs.

The Gumbel EVD states that the probability of occurring a score x greater than or equal to S is:

$$p(x \geq S) = 1 - e^{-\lambda(S-\mu)} \tag{S1}$$

Here λ is the scale parameter, and μ is the location parameter. Karlin and Altschul established a statistical theory about local alignment statistics [7], which makes five

assumptions:

1. At least one score is positive.

2. The expected score must be negative; otherwise the segment with the maximum score would tend to be the whole sequence.

3. The letters of the sequences are i.i.d.

4. The sequence lengths are sufficiently large.

5. No gaps are allowed within the local alignments.

Under such assumptions, Karlin and Altschul considered the statistical significance of local alignments between two independent random sequences consists of letters sampled independently from an alphabet of letters $A = a_1, a_2, \ldots, a_r$ with respective probabilities $p_1, p_2, \ldots, p_r$ and $p'_1, p'_2, \ldots, p'_r$.

Hence, the paring of a_i from the first sequence and a_j from the second sequence occurs with probability $p_i p'_j$. Let the score for such a paring be S_{ij}. By the assumption, there is some probability of a positive score and the expected pair score $\sum p_i p'_j S_{ij}$ is negative. Let real number λ^* be the unique positive solution to the equation

$$\sum_{i,j} p_i p'_j e^{\lambda S_{ij}} = 1 \tag{S2}$$

.

Karlin and Altschul proved that for large x,

$$\begin{aligned} Prob\left\{ M > \frac{\ln m_a n_a}{\lambda^*} + x \right\} &\leq K^* e^{-\lambda^* x} \\ &= e^{-\lambda^* \left(x - \frac{\ln K^*}{\lambda^*} \right)} \end{aligned} \tag{S3}$$

where K^*, like parameter λ^*, can also be computed from the set of scores and probabilities $p_1, p_2 2, \ldots, p_r$. m_a is the actual length of the query and n_a is the actual length of the database. From equation S3, one can identify the location parameter μ and the number of alignments expected by chance during a sequence database search, the E-value,

$$p(S \geq x) = 1 - e^{-E} \tag{S4}$$

Therefore,

$$E = e^{-\lambda(S-\mu)} = Kmne^{-\lambda S} \tag{S5}$$

which is the famous Karlin-Altschul equation.

Edge Effect Here, the fourth assumption, i.e., that the sequence lengths are sufficiently large for the asymptotic theory to be accurate, does not hold. Accordingly, we must replace the actual sequence lengths m and n within the theory by the "effective" lengths m' and n', where $m' = m - l$ and $n' = n - Nl$. They are introduced to compensate the edge effect of alignments [1], which occurs at the end of the query sequence or the database sequence, where there may not be enough sequence space to construct an optimal alignment.

Given the statistical parameters α, β, λ, and K, the edge effect parameter l may be found as the solution to the equation:

$$l = \frac{\alpha}{\lambda} \times \ln(Km'n') + \beta = \frac{\alpha}{\lambda} \times \ln\big(K(m-l)(n-Nl)\big) + \beta \tag{S6}$$

In large search spaces, l may be greater than m_a, resulting in a negative effective length m. In cases like this, if the effective length is shorter than $1/k$, then it is set to $1/k$ to cancel its contribution to the expect-value E.

The discussions above are all based on alignments that have no gaps [3]. For gap-alignments, BLAST algorithm computes the gap penalty, which consists of two parts, the gap existence penalty a and the gap extension penalty b. Thus a gap of k residues gets a total score of $-(a + bk)$. For alignments with gaps, there is no analytical formula for values of λ, K, and H.

Spouge Statistics

Finite size correction [5] was introduced since version 2.2.26. Instead of estimating l, FSC estimates

$$area = E[m - L_I(y)]^{+}[n - L_J(y)]^{+} \tag{S7}$$

as a measure of $(m - l)(n - Nl)$. Here, I, J are two sequences to be compared. $L_I(y)$ is the distribution of the length required to attain a score of y or more. Equation S7 is

practically computed by approximating the distribution of $\langle L_I(y), L_J(y)\rangle$. This is approximated by a bivariate normal distribution with means

$$l_I(y) = E[L_I(y)], l_J(y) = E[L_J(y)] \quad \text{(S8)}$$

variances

$$v_I(y) = var(L_I(y)), v_J(y) = var(L_J(y)) \quad \text{(S9)}$$

and covariance

$$c(y) = cov(L_I(y), L_J(y)) \quad \text{(S10)}$$

These parameters are estimated using linear functions of the score y using following formulas

$$\begin{aligned} l_I(y) &= a_I y + b_I, l_J(y) = a_J y + b_J \\ v_I(y) &= \alpha_I y + \beta_I, v_I(y) = \alpha_J y + \beta_J \\ c(y) &= \sigma y + \tau \end{aligned} \quad \text{(S11)}$$

$a_I = a_J = a$, $b_I = b_J = b$, $\alpha_I = \alpha_J = \alpha$, $\beta_I = \beta_J = \beta$, and σ are computed using a rigorous mathematical formula which depend on gap penalties. These values are precomputed for different scoring matrices. $b_I, b_J, \beta_I, \beta_J, and\ \tau$ is approximated from $\alpha, \beta, \sigma, a, b$.

These parameters don't depend on the length of the database or the query. However, in actual BLAST implementations using Spouge statistics, the formula is modified to include a database scale factor. The database scale factor is calculated using the formula,

$$db\_scale\_factor = \frac{n'}{m'} \quad \text{(S12)}$$

For a given HSP with score S, evalue is calculated using

$$E = area \times Ke^{-\lambda S} \times db\_scale\_factor \quad \text{(S13)}$$

Existing e-value correction software and their features

mpiBLAST

mpiBLAST [4] is a parallel implementation of NCBI BLAST on the cluster. It segments the database, ports the segments into different nodes of a cluster, and runs parallel BLAST search jobs against database segments on different nodes. Once the parallel search jobs return, it aggregates the search result. It has two important contributions. First, it achieves super-linear speedup by reducing IO overhead (time spent in reading and writing hard-disk storage). Second, it is the first parallel BLAST tool to provide exact e-value statistics in contrast to approximate e-value statistics of other contemporary parallel implementations of NCBI BLAST.

mpiBLAST's exact e-value statistics requires two steps. First, it collects the necessary statistical parameters for the entire database by performing a pseudo-run of the BLAST engine against the global database. Once it has the global parameters, it passes the global parameters (such as whole database length n, the total number of sequences N) to the parallel search jobs against segmented databases. mpiBLAST modifies some functionalities of NCBI BLAST (blast.c, blastdef.h, blastkar.c, and blastutl.c) so that global parameters can be fed externally and that information can be used to calculate exact e-values.

For accurate e-value correction, mpiBLAST requires prior knowledge of the entire database.

NOBLAST

NOBLAST [6] provides new options for NCBI BLAST. It offers a way to correct e-values when split databases are used and the results need to be aggregated. E-value computation requires knowledge about the entire database size, the number of sequences in the whole database N and the total length of the database n. Using the values N, n and Karlin-Altschul statistical parameters which are independent of database size, the e-value can be computed using Karlin-Altschul statistics. First, NOBLAST computes the length adjustment using the knowledge about the complete original database, then, it computes effective search space using length adjustment, and finally, it computes the e-value using effective search space.

In principle, NOBLAST takes a similar approach to mpiBLAST, as both provide global statistical parameters to the search jobs against a segmented database so that that exact e-value can be computed. While mpiBLAST's main contribution is a parallel implementation and e-value correction comes from the need of producing the same output as the sequential counterpart, NOBLAST's main contribution is an e-value correction. Both tools require prior knowledge about the entire database. Both tools were developed before Spouge's e-value statistics were introduced, so they didn't address e-value corrections for the BLAST programs that use Spouge's statistics.

e-value correction

We use algorithm S1 to recompute e-values for BLAST programs using Karlin-Altschul statistics. We first aggregate database sizes from two input results and use the aggregated size$N$ to compute length adjustment l. Using N and l, we recompute e-values for both results.

Algorithm S1 Recomputing e-values for Karlin-Altschul Statistics

```
Input: result1, result2
n ← result1.n + result2.n
m ← result1.m
N ← result1.N + result2.N
l ← recompute_length_adjustment(n, m, N)
recompute_evalues(result1, l, N)
recompute_evalues(result2, l, N)
```

We use algorithm S2 to re-scale e-values for BLAST programs using Spouge statistics. First we aggregate the database sizes for two input results, and scale the e-values by a factor of the ratio between aggregated database size and the individual database size.

Algorithm S2 Re-scale e-values for Spouge statistics

Input: $result1, result2$

$db\_length1 \leftarrow result1.db\_length$

$db\_length2 \leftarrow result2.db\_length$

$db\_length \leftarrow db\_length1 + db\_length2$

$re\text{-}scale\_evalues(result1, \frac{db\_length}{db\_length1})$

$re\text{-}scale\_evalues(result2, \frac{db\_length}{db\_length2})$

Algorithm to merge two search results through e-value correction

Algorithm S3 Merging results for Karlin-Altschul/Spouge statistics

Input: $result1, result2$

$merged\_result \leftarrow \Phi$

recompute/re-scale e-values

$m, n \leftarrow 0$

for $i = 1 \rightarrow num\_of\_hits$ **do**

  $e\text{-}value1, score1 \leftarrow min(result1.alignment[m].hsps)$

  $e\text{-}value2, score2 \leftarrow min(result2.alignment[n].hsps)$

  if ($e\text{-}value1 < e\text{-}value2$) or ($e\text{-}value1 == e\text{-}value2$ and $score1 > score2$) **then**

    $merged\_result.add(result1.alignments[m])$

    increment m

  else

    $merged\_result.add(result2.alignments[n])$

    increment n

  end if

end for

return $merged\_result$

Data collection for case studies II and III

To obtain the venom gland transcriptome, 15 venom glands were dissected from newly emerged adult females from wild-collected oak (oak spp.) hedgehog galls *Acraspis erinacei* and placed in RNAlater stabilization solution. Pooled tissues were homogenized

in lysis buffer using a Bead Ruptor 12 (Omni International) with additional lysis with a 26-gauge syringe. RNA was extracted from the sample using the RNaqueous Micro kit followed by DNase I treatment as specified by the kit and confirmed to be of good quality using the Bioanalyzer 2100 (Agilent). The Illumina HiSeq library was prepared from 200 ng RNA using the TruSeq Stranded mRNA kit and sequenced in 150 bp single-end reads across two Rapid Run lanes on the Illumina HiSeq 2500 (Penn State Genomics Core Facility, University Park, USA) along with nine other barcoded wasp samples.

Raw sequence data (30596191 reads, available at NCBI SRA achieve under Accession ID SRR14053705) quality was assessed using FastQC v0.10.0 (available at `http://www.bioinformatics.babraham.ac.uk/projects/fastqc/`), and appropriate trimming for Illumina single-end reads was conducted using Trimmomatic v0.35 [2] to remove adapters and low quality bases (ILLUMINACLIP:TruSeq3-SE:2:30:10 LEADING:20 TRAILING:20 MINLEN:50 AVGQUAL:20), which removed 0.12% of the total reads and *de novo* transcriptome assembly from these QC-passed trimmed reads($30,559,248$ in total) was performed on the Trinity RNA-Seq *de novo* Assembler (version: trinityrnaseq r20140717) [8]. The transcriptome assembly consists of $44,440$ transcripts with the contig N50 of 865 bases. Transdecoder v5.3.0 (available at `https://github.com/TransDecoder/TransDecoder/wiki`) was used to predict $17,927$ protein sequences which were used as queries for Case studies II and III.

iBLAST software allows the user to perform incremental BLAST search with minimal overhead

The iBLAST program v1.0 includes a collection of Python scripts that can be downloaded at `https://github.com/vtsynergy/iBLAST`. To facilitate the use of iBLAST, we have provided instructions for installation, a user's manual, a quick start guide and examples of how to use iBLAST in several typical scenarios of bioinformatics research.

First, the user needs to copy the source folder and run the following command from this directory to install iBLAST:

```
./iBLAST-installer.sh
```

Next, we move onto the Python scripts, as described below.

Main program: iBLAST.py This program provides intelligent and incremental BLAST search options. It takes in a regular BLAST search command and performs an incremental search. Below is an example of how to use the script.

```
python iBLAST.py "blastp -db nr -query Trinity-tx.fasta -outfmt 5 -out result.xml"
```

Merge scripts: These scripts merge the results of two BLAST searches in XML format and produce an XML output with corrected e-values.

1. **BlastpMergerModule.py:** This script merges the results obtained using Karlin-Altschul statistics (e.g., blastn results).

2. **BlastnMergerModule.py:** This script merges the results obtained using Spouge statistics (e.g., blastp results).

3. **BlastpMergerModuleX.py** These scripts merge more than two BLAST results. They require several results to merge the input and output.

```
python BlastpMergerModule.py input1.xml input2.xml output.xml
python BlastnMergerModule.py input1.xml input2.xml output.xml
python BlastpMergerModuleX.py 3 input1.xml input2.xml input3.xml output.xml
```

Data source for case study I

100 nucleotide sequences from *Bombus impatiens* are available at `ftp://ftp.ncbi.nlm.nih.gov/genomes/Bombus_impatiens/CHR_Un/bim_ref_BIMP_2.1_chrUn.fa.gz`. 100 protein sequences from *Bombus impatiens* assembly are available at `ftp://ftp.ncbi.nlm.nih.gov/genomes/Bombus_impatiens/protein/protein.fa.gz`.

Creating experimental databases

Pre-formatted BLAST databases such *nt* and *nr* come in incremental parts. With progression of time, new sequences are packaged in parts and added to the databases.

Databases for case study I

For case study I, we consider three time steps when the nt and nr databases had 30, 40, and 50 parts. For these three time periods, we construct three databases as instances of nt and nr by combining 30, 40, and 50 parts using BLAST tool *blastdb_aliastool*. The incremental databases between two periods are also constructed. Table S1 shows different instances of *nt* databases in three different periods.

Table S1. Incremental nt Databases for case study I.

Time Period	Database parts	Number of sequences	Number of bases	Longest sequence length (bases)
T_0	0-29	25,117,275	80,740,533,243	774,434,471
$T_0 \rightarrow T_1$	30-39	8,389,596	33,008,962,097	275,920,749
T_1	0-39	33,506,871	113,749,495,340	774,434,471
$T_1 \rightarrow T_2$	40-59	8,891,258	38,722,333,261	129,927,919
T_2	0-49	42,398,129	152,471,828,601	774,434,471

Table S2 shows different instances of *nr* databases in three different periods.

Table S2. Incremental nr databases for case study I

Time Period	Database parts	Number of sequences	Number of residues	Longest sequence length (residues)
T_0	0-29	49,468,463	17,686,779,866	36,507
$T_0 \rightarrow T_1$	30-39	15,878,318	6,065,300,773	35,523
T_1	0-39	65,346,781	23,752,080,639	36,507
$T_1 \rightarrow T_2$	40-49	16,448,075	6,278,067,810	38,105
T_2	0-49	81,794,856	30,030,148,449	38,105

Databases for case study II

We construct nr database instances for time 0 and 1 by combining 64 and 90 parts respectively. We combine these parts using *blastdb_aliastool*.

Table S3. Incremental nr databases for case study II

Time Period	Database	Number of sequences	Total residues	Longest sequence length (residues)
T_0	0-63	109,407,071	40,077,622,077	38,105
$T_0 \rightarrow T_1$	64-90	52,860,187	19,192,851,238	74,488
T_1	0-90	162,267,258	59,270,473,315	74,488

Fidelity of iBLAST

Table S4. Case study I: Fidelity of iBLAST in three consecutive time periods. *blastp* search was performed on nucleotide protein databases (nr). At any time instance, the *Past* database size is the size of the database from the previous time instance. The *Present* database size is the final database size at that instance. *Delta* is the incremental database growth from previous time instance to the current time instance. NCBI BLAST is performed on the *Present* database size, while iBLAST is performed only on *Delta*.

NCBI BLAST → Present; iBLAST → Delta

Time	Search	Data-base	Database Size		Delta = Present − Past	e-value Match	Hit Match
			Past	Present			
t_0	blastp	nr	0	17,686,779,866	17,686,779,866	100%	100%
t_1	blastp	nr	17,686,779,866	23,752,080,639	6,065,300,773	100%	100%
t_2	blastp	nr	23,752,080,639	30,030,148,449	6,278,067,810	100%	100%

Table S5. Case study II: Fidelity of iBLAST in two consecutive time periods. *blastp* search was performed on a large-scale novel transcriptome. At any time instance, the *Past* database size is the size of the database from the previous time instance. The *Present* database size is the final database size at that instance. *Delta* is the incremental database growth from previous time instance to the current time instance. NCBI BLAST is performed on the Present database size, while iBLAST is performed only on *Delta*.

NCBI BLAST iBLAST

Time	Search	Data-base	Database Size		Delta = Present − Past	e-value Match	Hit Match
			Past	Present			
t_0	blastp	nr	0	40,077,622,077	40,077,622,077	100%	100%
t_1	blastp	nr	40,077,622,077	59,270,473,315	19,192,851,238	100%	100%

Load-balancing via query partitioning

For case study II and III, we have partitioned 17927 queries into 20 query files based on number of residues after randomizing the order instead of a more straightforward partitioning based on number of queries while keeping the original order.

If we partition the queries by making sure each partition has roughly same number of queries without disrupting their order, we get a range of execution times demonstrating lack of proper load balancing. The standard deviation in iBLAST search times is 2748 seconds and standard deviation in NCBI BLAST search times is 8727 seconds. This means the compute nodes have to wait idly for $1 - 3$ hours on average. Fig S2 demonstrates the lack of load balancing.

In contrast, when we first randomize the order of the queries and then partition the queries by making sure that all partitions have the roughly same number of residues, the standard deviations fall to 150 and 487 seconds respectively (Fig S3).

Explanation for NCBI BLAST missing many top hits

Due to the early cutoff of max target sequence used by its heuristic algorithm. NCBI BLAST performs search in two phases. In earlier phase (ungapped extension), it starts with matching a seed sub-string between target and query sequence and then extends

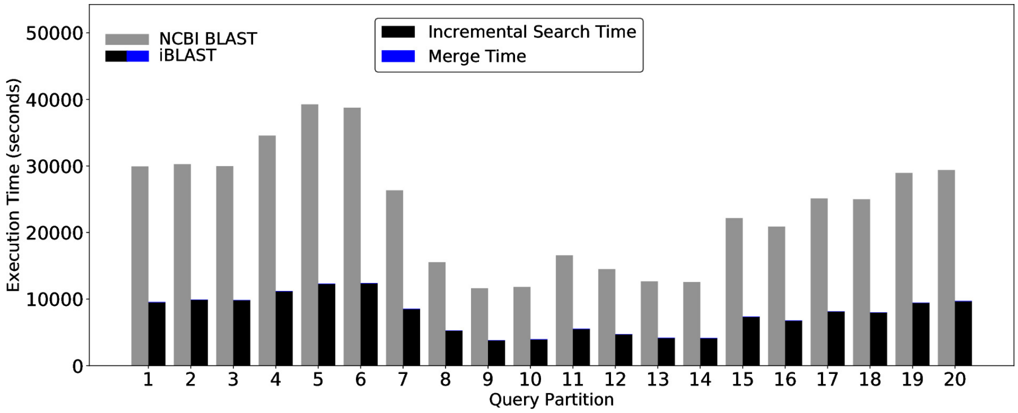

Fig S2. Load imbalance resulted from a naive query partitioning. Execution time when a straightforward query partitioning scheme is adopted, which results in significant lack of load balancing. The standard deviation for the execution times for both incremental and NCBI BLAST searches are large (2748 and 8727 seconds respectively).

the matching pair in both direction without allowing any gap. In this phase, BLAST algorithm assigns some scores to these matching pairs and keeps only the very high scoring pairs using a cutoff determined by e-value cutoff or number of maximum hits. In the gapped phase, these selected high scoring pairs are further extended in both directions while allowing gaps and these evolved pairs get changed scores. Some of the pairs that did not make the cut during the ungapped extension, can become high scoring pairs. For a larger database, these missed opportunities are higher in number because there are more potential pairs in the ungapped phase. Since iBLAST is combining results from smaller databases, it misses relatively smaller number of those high scoring hits compared to NCBI BLAST.

Computing delta database

A formatted NCBI database consists of several index files and actual sequences are partitioned into several 1GB partitions. For example non redundant protein database *nr* has 100 1GB partitions named as nr.00, nr.01, ..., nr.99. While saving an instance of the database, AdaBLAST saves a list of these file names. Let, at time t_1, database db had P_{t_1} parts, so the *Record Database* will save the instance by saving the list $L_1 = db.00, db.01, \ldots, db.(P_{t_1} - 1)$. At time t_2, nr got updated and now it has P_{t_2} data files. The instance of db at time t_2 is the list $L_2 = db.00, db.01, \ldots, db.(P_{t_2} - 1)$. To compute delta database between times t_1, t_2, the *Incremental Logic* module will first

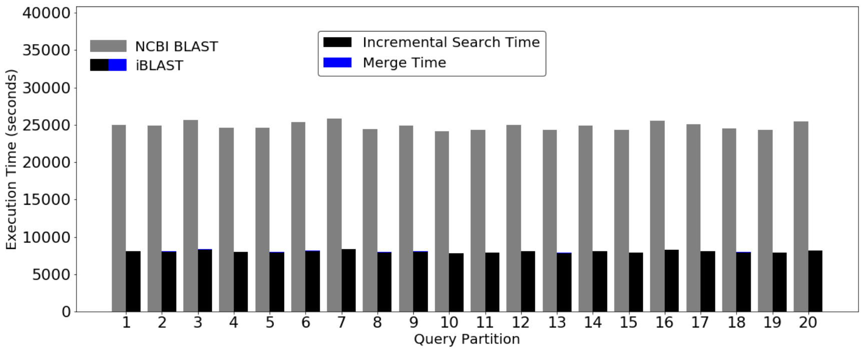

Fig S3. Load balance demonstrated by our proposed strategy. Execution time when our improvised query partitioning scheme is adopted which results in better load balancing. The standard deviation for the execution times for both incremental and NCBI BLAST searches are minimal compared to the naive strategy (150 and 487 seconds respectively).

compute the difference between the two lists saved as the two instances. The difference is $\delta L = L_2 - L_1 = db.(P_{t_1}), \ldots, db.(P_{t_2} - 1)$. We then use *blastdb_aliastool* distributed with command-line BLAST to make the delta database using the files in δL.

There is one caveat, the last file $(db.(P_{t_1} - 1))$ in the instance at t_1 might have been updated with new sequences and by not including them, we might miss some of the potential hits. To mitigate this effect, we include $db.(Pt_1 - 1)$ in the delta database. When recomputing or re-scaling the e-values, we use the correct previous and current database sizes (Fig S4).

It takes few milliseconds to compute the delta database from two instances.

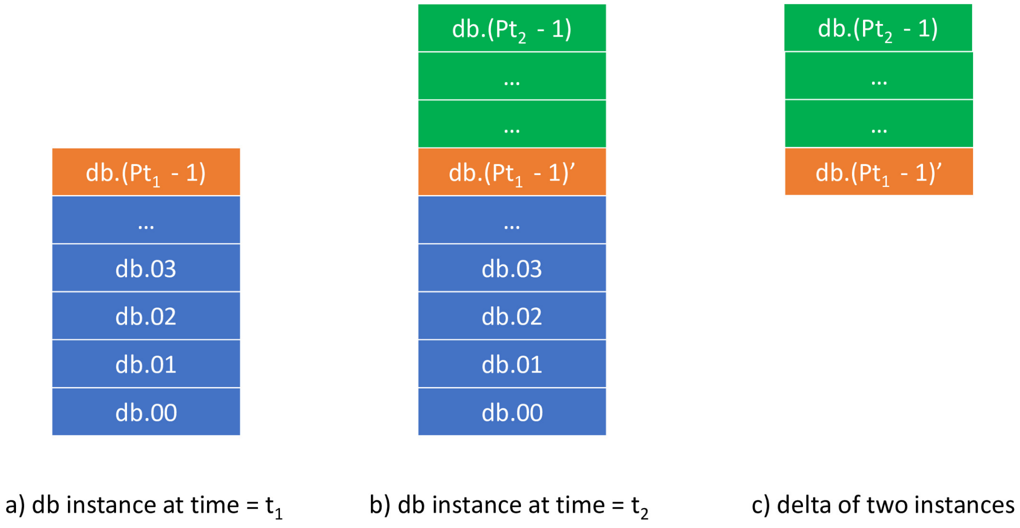

Fig S4. Delta database computation from two instances of the same database *db*. At time t_1, the instance has Pt_1 parts. At time t_2, the instance has Pt_2 parts. In both of these instances, the part $db.(Pt_1)$ is common. However this part is potentially updated. So, we take all the parts from $db.(Pt_1)$ to $db.(Pt_2)$ to compute the delta database.

References

1. Altschul SF, Gish W, et al. Local alignment statistics. Methods in enzymology. 1996;266(2):460–480.

2. Bolger AM, Lohse M, Usadel B. Trimmomatic: a flexible trimmer for Illumina sequence data. Bioinformatics. 2014;30(15):2114–2120.

3. Altschul SF, Gish W, Miller W, Myers EW, Lipman DJ. Basic local alignment search tool. Journal of molecular biology. 1990;215(3):403–410.

4. Darling AE, Carey L, Feng WC. The design, implementation, and evaluation of mpiBLAST. Los Alamos National Laboratory; 2003.

5. Park Y, Sheetlin S, Ma N, Madden TL, Spouge JL. New finite-size correction for local alignment score distributions. BMC research notes. 2012;5(1):286.

6. Lagnel J, Tsigenopoulos CS, Iliopoulos I. NOBLAST and JAMBLAST: New Options for BLAST and a Java Application Manager for BLAST results. Bioinformatics. 2009;25(6):824–826.

7. Karlin S and Altschul SF. Methods for assessing the statistical significance of molecular sequence features by using general scoring schemes. Proceedings of the National Academy of Sciences. 1990;87(6):2264–2268.

8. Grabherr MG, Haas BJ, Yassour M, Levin JZ, Thompson DA, Amit I, et al. Full-length transcriptome assembly from RNA-Seq data without a reference genome. Nature biotechnology. 2011;29(7):644.
